# High-Bandwidth Repetitive Trajectory Tracking Control of Piezoelectric Actuators via Phase–Hysteresis Hybrid Compensation and Feedforward–Feedback Combined Control

**DOI:** 10.3390/mi14112009

**Published:** 2023-10-29

**Authors:** Jie Yuan, Haitao Wu, Yanding Qin, Jianda Han

**Affiliations:** 1 Institute of Intelligence Technology and Robotic Systems, Shenzhen Research Institute of Nankai University, Shenzhen 518083, China; 2120220532@mail.nankai.edu.cn (J.Y.); 1120220223@mail.nankai.edu.cn (H.W.); hanjianda@nankai.edu.cn (J.H.); 2 State Key Laboratory of Precision Electronic Manufacturing Technology and Equipment, Guangzhou 510006, China; 3 College of Artificial Intelligence, and Engineering Research Center of Trusted Behavior Intelligence, Ministry of Education, Nankai University, Tianjin 300350, China

**Keywords:** hysteresis compensation, trajectory tracking, direct inverse modeling, rate dependence, high frequency

## Abstract

Piezoelectric actuators (PEAs) are widely used in many nano-resolution manipulations. A PEA’s hysteresis becomes the main factor limiting its motion accuracy. The distinctive feature of a PEA’s hysteresis is the interdependence between the width of the hysteresis loop and the frequency or rate of the control voltage. Generally, the control voltage is first amplified using a voltage amplifier (VA) and then exerted on the PEA. In this VA-PEA module, the linear dynamics of the VA and the nonlinearities of the PEA are coupled. In this paper, it is found that the phase lag of the VA also contributes to the rate dependence of the VA-PEA module. If only the PEA’s hysteresis is considered, it will be difficult to achieve high-frequency modeling and control. Consequently, great difficulties arise in high-frequency hysteresis compensation and trajectory tracking, e.g., in the fast scanning of atomic force microscopes. In this paper, the VA-PEA module is modeled to be the series connection of a linear subsystem and a nonlinear subsystem. Subsequently, a feedforward phase–dynamics compensator is proposed to compensate for both the PEA’s hysteresis and the phase lag of the VA. Further, an unscented Kalman-filter-based proportional–integral–derivative controller is adopted as the feedback controller. Under this feedforward–feedback combined control scheme, high-bandwidth hysteresis compensation and trajectory tracking are achieved. The trajectory tracking results show that the closed-loop trajectory tracking bandwidth has been increased to the range of 0–1500 Hz, exhibiting excellent performance for fast scanning applications.

## 1. Introduction

Hysteresis modeling and compensation is one of the most popular topics in the motion control of piezoelectric actuators (PEAs) [1,2,3]. The measured hysteresis of a PEA exhibits strong rate dependence [4], i.e., the width of the hysteresis loop becomes wider with an increment in the input frequency (or input rate). Modeling of and compensation for the rate-dependent hysteresis have become one of the challenges in implementations, especially in high-frequency applications.

The generation of a PEA’s hysteresis is very complex, making physical modeling very challenging. Currently, no accurate and widely applicable physical hysteresis model is reported. As a result, phenomenological hysteresis models are widely adopted. Popular hysteresis models include the Preisach model [5], Prandtl–Ishlinskii (PI) model [6,7,8], and Bouc–Wen model [9], etc. The PEA’s hysteresis exhibits obvious rate dependence and saturation properties. As a result, modifications have been proposed to improve the modeling accuracy of these hysteresis models. For instance, Qin et al. proposed using a polynomial as the saturation operator and modeling the rate dependence by linearly varying the weights of the backlash operators according to the input rate [10]. Janaideh et al. utilized a stop operator to account for the saturation property [11]. Zhu et al. proposed a Gaussian process-based model capable of describing the rate dependence [4]. Liu et al. proposed a dynamic linearized neural network model which can precisely predict the output of the PEA at a frequency range of 0–200 Hz [12].

Repetitive trajectory tracking of PEAs is very important in scanning-based tasks such as atomic force microscopy (AFM) [13,14,15]. For instance, an amplitude and phase estimation method based on the acquisition of four points per oscillation was proposed for repetitive control of AFM [16]. Kalman and Lyapunov filters were also used to estimate the amplitude and phase [17,18]. Equally, directional repetitive control was proposed to align the scanning direction with the sample orientation [15]. A higher frequency is desired in these applications to accelerate the manipulation. However, great difficulties arise in tracking high-frequency trajectories because the rate dependence will become severe at high frequencies. Moreover, the bandwidth of the VA is also a bottleneck. Currently, the closed-loop trajectory tracking bandwidth mainly lies at the level of 200~300 Hz [4,19]. For instance, in our previous work, 200 Hz trajectory tracking was achieved by using an adaptive Kalman filter to dynamically update the parameters of the hysteresis compensator [20]. Active disturbance rejection control and current cycle iterative learning control were also used in the PEA’s high-frequency trajectory tracking [21].

Typically, PEAs are used together with voltage amplifiers (VAs). The VA can be treated as linear within its bandwidth. However, the VA’s linear dynamics and the PEA’s hysteresis are coupled. In this paper, it has been found that the phase lag of the VA also contributes to the rate dependence of the hysteresis of the VA-PEA module, especially at higher frequencies. Therefore, the phase lag of the VA-PEA module should also be compensated for if high-bandwidth manipulation is pursued.

In this paper, a feedforward phase–dynamics compensator is proposed for the VA-PEA module, where the hysteresis of the PEA is compensated for using an inverse PI model, and the phase lag of the VA-PEA module is compensated for using a lead compensator. In order to further account for the modeling uncertainties and improve the motion accuracy, a feedforward–feedback combined control scheme is also established, where an unscented Kalman filter (UKF)-based proportional–integral–derivative (PID) controller is cascaded in the feedback loop. Tracking of the sinusoidal and triangular trajectories is implemented, and a closed-loop trajectory tracking bandwidth of 0–1500 Hz is achieved.

## 2. High-Bandwidth Hysteresis Dynamics Hybrid Modeling of the VA-PEA Module

### 2.1. Characteristics of the Measured Hysteresis of the VA-PEA Module

**Figure 1:**
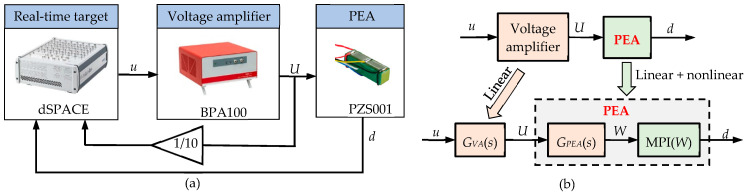
(**a**) Architecture of the VA-PEA module and (**b**) schematic diagram of the VA-PEA module, where *G_VA_* (*s*) represents the linear dynamics of the VA, and the rate-dependent hysteresis of the PEA is represented by a linear dynamics *G_PEA_* (*s*) and a PI model *H* (*U*).

The typical signals involved in a VA-PEA module are schematically illustrated in Figure 1. The control signal *u* generated by the real-time target (the control unit), and is amplified by the VA. The amplified voltage *U* is then exerted on the PEA. The output displacement of the PEA is denoted as *d*. In practice, the control voltage *u* is measured instead of *U* in hysteresis modeling. In this case, the VA-PEA module is considered a single unit, as shown Figure 1. As a result, the VA’s dynamics and the PEA’s hysteresis are coupled in the measurement [22]. The identified hysteresis model actually shows the relationship of *u*→*d*, i.e., *u*→*U*→*d*. According to Figure 1, the dynamics of the VA are totally included in the hysteresis model, which introduces unwanted uncertainties into the hysteresis model and significantly increases the difficulty and complexity.

In this paper, to separate the VA’s dynamics from the PEA’s hysteresis in the measurements, a BPA100 from Thorlabs is used as the VA as it can provide the monitoring voltage of the amplified voltage *U*. The PEA (PZS001 from Thorlabs) is adopted and its displacement is measured using a Wheatstone bridge amplifier. This PEA is a co-fired stack actuator with strain gauges. The maximum displacement is 17.4 μm at a maximum driving voltage of 150 V. Its size is 7 mm × 6 mm × 20 mm, and the operating temperature range is −25 to 85 °C. The data acquisition and closed-loop control algorithm are implemented on a real-time target (microlabbox from dSPACE) at a sampling rate of 25 kHz.

**Figure 2:**
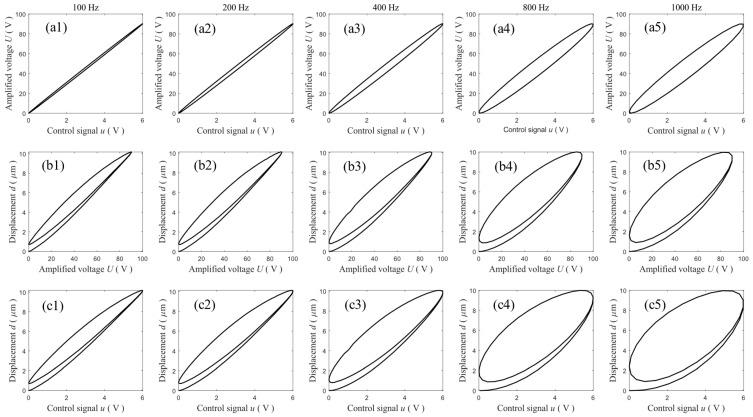
The measured input–output relationships of the VA-PEA module actuated by sinusoidal signals at 100 Hz, 200 Hz, 400 Hz, 800 Hz, and 1000 Hz. (**a1**–**a5**) the *u*→*U* relationships, (**b1**–**b5**) the *U*→*d* relationships, and (**c1**–**c5**) the *u*→*d* relationships.

The hysteresis loop is special as it only shows the input–output relationship. The responses of the VA-PEA module under sinusoidal signals are shown in Figure 2. Figure 2(b1–b5) show the hysteresis loops of the PEA, i.e., the *U*→*d* relationship. The rate dependence can be easily observed. Figure 2(a1–a5) show the measured *u*→*U* curves, where hysteresis-like loops also appear. Moreover, similar rate dependence can be observed in the *u*→*U* curves. It must be pointed out that in conventional VA-PEA setups, *U* is generally not recorded. In this case, the *u*→*d* relationship is recorded and utilized in hysteresis compensation. Figure 2(c1–c5) show the *u*→*d* relationship of the VA-PEA module. Obviously, as the VA’s dynamics is also included, more severe rate dependence is observed.

### 2.2. The Hysteresis-like Loops of a Linear Dynamic System

The measured input–output relationships in Figure 2 clearly show that hysteresis-like loops also exist in linear systems. Without loss of generality, a second-order linear system with the following transfer function is adopted as an example to investigate the hysteresis-like loops of linear systems:

(1) $$G(s)=\frac{k\cdot \omega_{n}^{2}}{s^{2}+2\zeta \omega_{n}s+\omega_{n}^{2}}$$

where *G*(*s*) is the transfer function showing the relationship between the Laplace transform of the output to the Laplace transform of the input, *s* is the complex frequency, and *k*, *ω_n_*, and *ζ* are the gain, natural frequency, and damping ratio, respectively.

**Figure 3:**
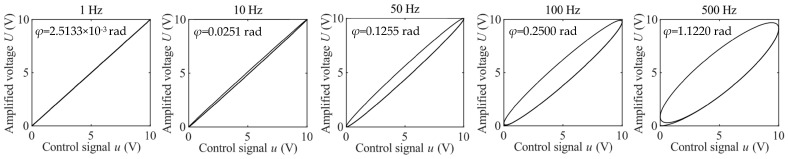
Hysteresis-like loops and phase lags of the second-order linear system at different frequencies.

Without loss of generality, the following values are adopted: *k* = 1, *ω_n_* = 5000 rad/s, and *ζ* = 1. The control signal is assumed to be a sinusoidal signal with an amplitude of 10 V. Figure 3 shows the input–output curves of this linear system at different frequencies. It can be observed that hysteresis-like loops also exist for this linear system. At low frequencies, e.g., 1 Hz, the phase lag *φ* is negligible and the hysteresis loop is not obvious. However, with an increment in frequency, the phase lag increases and the hysteresis loop becomes very wide. The results in Figure 3 are similar to the results in Figure 2(a1–a5). This implies that the VA’s dynamics can be well captured using a simple transfer function.

### 2.3. Hysteresis Dynamics Hybrid Modeling of the VA-PEA Module

Based on the above measurement and analysis, in a VA-PEA module, if the dynamics of the VA is included in hysteresis modeling, more modifications to the hysteresis model are inevitable and the effectiveness of the hysteresis model is limited within a small frequency bandwidth. This might not be a problem if the VA-PEA module works in low-frequency applications. However, if high-frequency manipulations are pursued, the dynamics of the VA cannot be ignored.

As shown in Figure 1b, the VA-PEA module can be treated as the series connection of a linear subsystem and a hysteretic subsystem. According to the above experimental data and analysis, the linear dynamics of the VA is assumed to be a second-order system *G_VA_*(*s*), which can be formulated to be:

(2) $$G_{VA}(s)=\frac{k_{1}\omega_{n1}^{2}}{s^{2}+2\zeta_{1}\omega_{n1}s+\omega_{n}{}_{1}^{2}}$$

where *k*_1_, *ω_n_*_1_, and *ζ*_1_ are the gain, natural frequency, and damping ratio, respectively.

For the hysteresis of the PEA, its rate dependence can be modeled in different ways [20,21,23]. As high-frequency manipulation is pursued herein, the combination of a linear dynamics *G_PEA_*(*s*) and a rate-independent PI model MPI(*W*) is adopted to model the PEA’s hysteresis. This helps to better compensate for the influence of the VA’s phase lag and the rate dependence. As illustrated in Figure 1b, *W*, an intermediate variable without physical meaning, is adopted to facilitate the modeling process. In this paper, a modified PI model is adopted to construct MPI(*W*), where a polynomial operator is cascaded to the classical PI model to account for the saturation (asymmetry) of the hysteresis loop. The classical PI model can be defined as follows:

(3) $$x=H_{\mathrm{PI}}(W)=\sum_{i=1}^{n}w_{hi}\cdot h_{i}(W)$$

where H(*W*) denotes the classical PI model, *n* is the order of the PI model, *w_hi_* is the weight, and *h_i_*(*W*) is the elementary backlash operator, which is defined in the following equation:

(4) $$h_{i}(W_{t})=\max\left\{W_{t}-r_{i},\;\min\left[W_{t}+r_{i},\;h(W_{t-T})\right]\right\}$$

where *r_i_* is the threshold and *T* is the sampling period. For the PEA, the backlash operator can be initialized as follows:

(5) $$h_{i}(W_{0})=\max\left\{W_{0}-r_{i},\;\min\left[W_{0}+r_{i},\;0\right]\right\}$$

Due to the symmetry of the backlash operators, the PI model is also symmetric about the loop center. To better fit the saturation (asymmetry) of the actual hysteresis loop, the following polynomial is adopted to morph the loop shape of the classical PI model:

(6) $$d=\mathrm{MPI}(W)=sat\left(H_{\mathrm{PI}}(W)\right)=c_{2m-1}x^{2m-1}+c_{2m-3}x^{2m-3}+\cdots +c_{1}x^{1}$$

where *d* is the displacement output of the VA-PEA module, *x* is the output of the classical PI model, and (2 *m* − 1) is the order of the polynomial. Based on our previous experience, it is found that odd order terms in the polynomial help to improve the accuracy of the modified PI model. Therefore, only the odd order terms are maintained in the saturation operator. Equations (3) and (6) formulate the modified PI model.

The rate dependence of the PEA’s hysteresis can be modeled using another second-order linear dynamics, as formulated below:

(7) $$G_{PEA}(s)=\frac{k_{2}\omega_{n2}^{2}}{s^{2}+2\zeta_{2}\omega_{n2}s+\omega_{n2}^{2}}$$

where *k*_2_, *ω_n_*_2_, and *ζ*_2_ are the gain, natural frequency, and damping ratio, respectively.

### 2.4. Parameter Identification

In parameter identification, the linear dynamics model of the VA and the rate-dependent hysteresis of the PEA are identified individually, as described below:

**(1) Identification of the VA’s linear dynamics.** In order to obtain a precise dynamics model across a wide frequency range, a 1~1000 Hz swept sinusoidal signal is used as the control voltage, and both *U* and *d* are recorded. Based on the measured *u* and *U*, the transfer function of the VA is identified and given below:

(8) $$G_{VA}(s)=\frac{2.0078\times 10^{14}}{s^{2}+1.2899\times 10^{8}s+1.3445\times 10^{13}}$$

**Figure 4:**
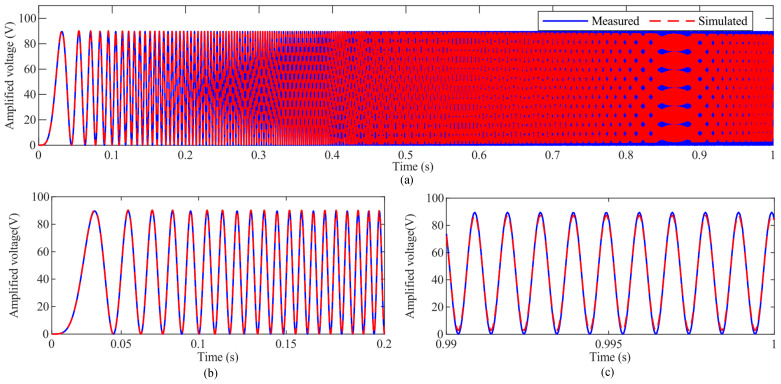
Measured and estimated amplified voltages of the VA: (**a**) the overall results, (**b**,**c**) the zoomed-in details from 0 to 0.2 s and from 0.99 to 1.0 s, respectively.

Both the measured *U* and the estimated results using Equation (8) are provided in Figure 4. It can be found that the estimated results fit the measurement well and only a slight discrepancy can be observed around 1000 Hz. The modeling accuracy is calculated to be 97.46%, and the root mean square error (RMSE) is 0.6322 V.

**(2) Identification of the PEA’s hysteresis**. As shown in Figure 1b, the PEA’s hysteresis is modeled into a series connection of *G_PEA_*(*s*) and MPI(*W*). After a trial and error process, it is found that a higher modeling accuracy can be obtained if *G_PEA_*(*s*) and MPI(*W*) are identified individually.

**Figure 5:**
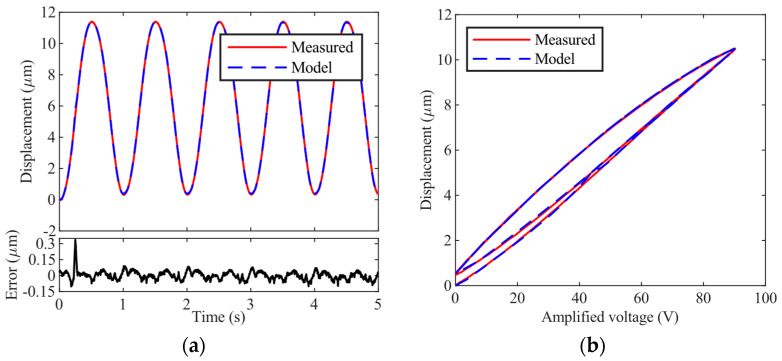
Comparison between the measurements and the identified modified PI model. (**a**) Time plot; (**b**) hysteresis plot.

The modified PI model MPI(*W*) is identified first. Because MPI(*W*) is defined as a rate-independent model, measurements under the actuation of a 1 Hz sinusoidal signal are used in identification. In this case, the rate dependence can be ignored, i.e., *G_PEA_*(*s*) can be simplified to be 1. Based on previous experience, both the orders of the PI model and the polynomial operator are set to be 10. The parameter identification results are listed in Table 1. The measured displacement and model output are shown in Figure 5.

For *G_PEA_*(*s*), *U* and the intermediate variable *W* are defined as the input and output, respectively. To obtain the intermediate variable *W* from the measured displacement *d*, it is necessary to obtain the inverse PI model, i.e., the *d*→*W* relationship. Following the direct inverse modeling approach established in our previous work [24], the inverse PI model can be directly identified from the measurements and is also of PI type. Therefore, the same model structure of the modified PI model formulated in Equations (3) and (6) is adopted to construct the inverse PI model. Similarly, the same measurements with the 1 Hz sinusoidal signal are used to identify the inverse PI model. The identified parameters of the inverse PI model are listed in Table 2 with a superscript of −1.

For the identification of *G_PEA_*(*s*), the same measurements under the 1~1000 Hz swept sinusoidal signal are utilized so as to include more rate dependence. The intermediate variable *W* is calculated using the inverse PI model. Subsequently, parameter identification of *G_PEA_*(*s*) is finished and the identification results are given below:

(9) $$G_{PEA}(s)=\frac{5.7042\times 10^{14}}{s^{2}+2.9345\times 10^{8}s+5.8060\times 10^{14}}$$

**Figure 6:**
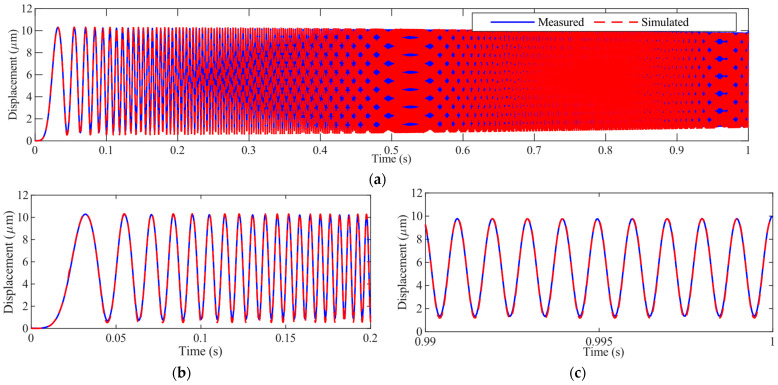
Measured and estimated displacement of the PEA: (**a**) the overall results, (**b**,**c**) the zoomed-in details from 0 to 0.2 s and from 0.99 to 1.0 s, respectively.

The measured displacement *d* and the model output are plotted in Figure 6. It can be observed that the identified rate-dependent hysteresis model can well predict the displacement of the VA-PEA module in a frequency range of 0~1000 Hz. The modeling accuracy is calculated to be 94.15%, and the RMSE is 36.31 nm.

## 3. Phase–Hysteresis Hybrid Compensation Strategy

As graphically illustrated in Figure 1b, the VA-PEA module can be treated as the integration of linear and nonlinear parts. Based on the hysteresis–dynamics hybrid modeling results in Section 2, a high modeling accuracy has been achieved across the frequency range of 0~1000 Hz. Therefore, one straightforward hysteresis compensation strategy is to cascade the inversion of the hysteresis–dynamics model to the VA-PEA module. In many conventional hysteresis compensation methods, only hysteresis compensation at relatively lower frequencies is pursued. In this case, the influence of the linear part is not very obvious, and thus only the nonlinear part of the system is considered. However, the hysteresis compensation performance at higher frequencies is not satisfactory.

**Figure 7:**
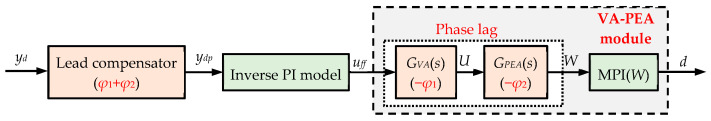
Schematic diagram of the phase–hysteresis hybrid compensation strategy, where *y_d_* and *y_dp_* are the desired trajectory and the phase-compensated desired trajectory, respectively.

Based on our preliminary tests, at higher frequencies, the influences of *G_VA_*(*s*) and *G_PEA_*(*s*) mainly come from their phase lags. Therefore, this paper proposes a phase–hysteresis hybrid compensation strategy. As shown in Figure 7, a lead compensator is used to add a phase lead to the desired trajectory *y_d_*, thus compensating for the phase lags of *G_VA_*(*s*) and *G_PEA_*(*s*). Subsequently, an inverse PI model is used to compensate for the hysteresis of the PEA. In this phase–hysteresis hybrid compensation, the displacement *d* can follow the desired trajectory *y_d_*.

**Figure 8:**
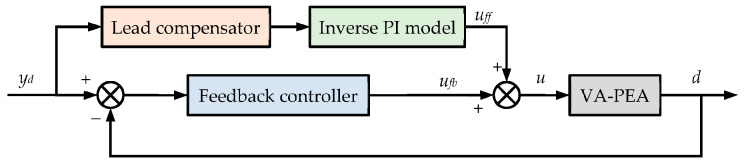
Schematic diagram of the feedforward–feedback combined control.

It must be pointed out that the hysteresis compensation performance of the above phase–hysteresis hybrid compensation highly depends on the modeling accuracy of the linear dynamics models and the inverse PI model. Further, feedforward compensation cannot account for the disturbances. Therefore, a feedforward–feedback combined control is proposed, as schematically presented in Figure 8. The majority of the influence of the phase lag and hysteresis is compensated for by the phase–hysteresis hybrid compensation in the feedforward loop. The feedback controller is only responsible for the modeling uncertainties. Detailed information on the proposed feedforward–feedback combined control is presented in the following subsections.

### 3.1. Lead Compensator and Inverse PI Model

In repetitive trajectory tracking, the typical desired trajectories include sinusoidal and triangular trajectories. For these periodic trajectories, the phase lags of *G_VA_*(*s*) and *G_PEA_*(*s*) can be calculated according to the frequencies. Based on the transfer functions of *G_VA_*(*s*) and *G_PEA_*(*s*) formulated in Equations (2) and (7), the corresponding phase lags can be calculated using the following formulas:

(10) $$\varphi_{1}(\omega )=\arctan\left(\frac{2\zeta_{1}\eta_{1}}{1-\eta_{1}^{2}}\right),\;\varphi_{2}(\omega )=\arctan\left(\frac{2\zeta_{1}\eta_{2}}{1-\eta_{2}^{2}}\right)$$

where *ω* is the frequency of the desired trajectory, and *η*_1_ = *ω*/*ω_n_*_1_ and *η*_2_ = *ω*/*ω_n_*_2_ are the ratios of the frequency to the resonant frequencies of *G_VA_*(*s*) and *G_PEA_*(*s*), respectively.

Basically, the lead compensator is equivalent to shifting the desired trajectory to the left along the time axis. The time shift of the lead compensator can be calculated using

(11) $$\Delta t=\frac{\varphi_{1}+\varphi_{2}}{\omega }$$

If the desired trajectory is a sinusoidal trajectory, it can be formulated into the following form:

(12) $$y_{d}(t)=A\sin(\omega t-\pi /2)+A$$

where *A* is the amplitude of the sinusoidal trajectory, and an initial phase of −π/2 is adopted such that the desired trajectory starts from the minimum position.

The time shift calculated in Equation (11) can be used to form the lead compensator. The compensated desired trajectory can be expressed as follows:

(13) $$\begin{array}{ll} y_{dp}(t) & =A\sin\left(\omega (t+\Delta t)-\pi /2\right)+A\\ & =A\sin\left(\omega t-\pi /2+\varphi_{1}+\varphi_{2}\right)+A \end{array}$$

Similarly, the triangular desired trajectory can be formulated into the following form:

(14) $$y_{d}(t)=2A\times \left|\frac{t}{T}-\mathrm{floor}(\frac{t}{T}+\frac{1}{2})\right|$$

where *A* is the amplitude of the triangular trajectory, and *T* is the period of the triangular trajectory. If the lead compensator is considered, the compensated desired trajectory can be expressed as follows:

(15) $$y_{dp}(t)=2A\times \left|\frac{t+\Delta t}{T}-\mathrm{floor}(\frac{t+\Delta t}{T}+\frac{1}{2})\right|$$

The identification of the inverse PI model was finished in Section 2.4, and thus the identification process will not be repeated herein. Based on our previous experience, the 10th-order PI model and the 10th-order polynomial operator are adequate to guarantee the modeling accuracy of the inverse PI model. The identified parameters of the inverse PI model are given in Table 2.

### 3.2. UKF-Based PID Controller

Many feedback controllers have been proposed in the hysteresis compensation of PEAs during the past decade [20,25,26]. In this paper, the feedback controller is only responsible for the modeling uncertainties. Therefore, the widely used PID controller is selected as the feedback controller. Further, due to the high modeling accuracy of the inverse PI model, the modeling uncertainties can be treated as slowly varying signals. In this case, the integral gain is more important as it affects the steady-state error of the system. In this paper, the derivative gain can be set to zero so as to simplify the parameter tuning. According to our preliminary tests, the integral gain varies between 100 and 2000. In order to further improve the consistency of the hysteresis compensation performances in tracking different trajectories, a UKF is used to dynamically tune the integral gain. In this manner, the hysteresis compensation performance can be maintained at both low and high frequencies.

The error *e* between the desired trajectory *y_d_* and the actual displacement *d* is *e* = *y_d_* − *d*. In this paper, the integral gain in the PID controller is defined as the state of the UKF, i.e., *x*. *F* and *H* are the state update function and measurement function, as described below:

(16) $$\begin{array}{l} x_{k+1}=F(x_{k})+U_{k}=x_{k}+U_{k}\\ u_{k+1}=H(x_{k})+V_{k}=K_{p}e_{k}+x_{k}\overset{k}{\underset{i=1}{\Sigma }}e_{i}+V_{k} \end{array}$$

where *U_k_* and *V_k_* are the process noise and observed noise, respectively, and *u_k_*_+1_ is the estimated control voltage.

The UKF proceeds as follows:

The first step is to find the mean *x*_k|k−1_ and variance *P* _k|k−1_ of the target state prediction based on the sigma points ***χ***_k−1_ and two weights $W_{i}^{m}$ and $W_{i}^{c}$:

(17) $$\left\{ \begin{array}{l} \chi_{k-1}={\left[x_{k-1}\cdots x_{k-1}\right]}_{l\times (2l+1)}-\left[\begin{array}{ccc} 0_{l\times 1} & -\sqrt{(l+\lambda )P_{k-1|k-1}} & \sqrt{(l+\lambda )P_{k-1|k-1}} \end{array}\right]\\ W_{0}^{m}=\frac{\lambda }{l+\lambda }\\ W_{0}^{c}=\frac{\lambda }{l+\lambda }+(1-\sigma^{2}+\vartheta )\\ W_{i}^{m}=W_{i}^{c}=\frac{1}{2(l+\lambda )},i=1\sim 2l \end{array}\right.$$

where *l* is the state dimension (here it is 1); *λ* is the constant, and *λ* = *σ^2^*(*l* + *κ*); *κ* is the scale factor and its value only needs to ensure that the covariance matrix is non-negatively definite; *σ* controls the range of sampling points’ distribution; and the tuning of *ϑ* can improve the approximate accuracy of the covariance.

The tuning of the parameters is straightforward. The range of *σ* is 10^−4^ ≤ *σ* ≤ 1. A larger *σ* value helps to increase the noise suppression, whereas the robustness might become weaker. As a result, *σ* is usually set to be a smaller value. For Gaussian distribution, *ϑ* can usually be set to 2. Based on related research [27,28], *κ* + *n* = 3, *σ* = 10^−3^, and *ϑ* = 2 are adopted in this paper. **$\chi_{1}^{k|k-}$** is the matrix after sampling expansion. The mean and variance of the target state prediction are:

(18) $$\left\{ \begin{array}{l} \chi_{k|k-1}=F(\chi_{k-1})\\ x_{k|k-1}=\sum_{i=0}^{2l}W_{i}^{m}\cdot \chi_{k|k-1}^{i}\\ P_{x(k|k-1)}=\sum_{i=0}^{2l}W_{i}^{c}(\chi_{k|k-1}^{i}-x_{k|k-1}){\left(\chi_{k|k-1}^{i}-x_{k|k-1}\right)}^{T}+Q_{k} \end{array}\right.$$

where *Q_k_* is the covariance matrix of the process noise.

The second step is to find the variance *S_k_* and *Ψ_k_* of the target observation prediction:

(19) $$\left\{ \begin{array}{l} \gamma_{\chi (k|k-1)}=H(\chi_{k|k-1})\\ y_{k|k-1}=\sum_{i=0}^{2l}W_{i}^{m}\gamma_{k|k-1}^{i}\\ S_{k|k-1}=\sum_{i=0}^{2l}W_{i}^{c}(\gamma_{\chi_{k|k-1}}^{i}-y_{k|k-1}){\left(\gamma_{\chi_{k|k-1}}^{i}-y_{k|k-1}\right)}^{T}+R_{k}\\ \psi_{k|k-1}=\sum_{i=0}^{2l}W_{i}^{c}(\chi_{k|k-1}^{i}-x_{k|k-1}){\left(\gamma_{k|k-1}^{i}-y_{k|k-1}\right)}^{T} \end{array}\right.$$

where *R_k_* is the covariance matrix of the measurement noise.

The third step is to update the mean and variance of the target state:

(20) $$\left\{ \begin{array}{l} x_{k|k}=x_{k|k-1}+\psi_{k|k-1}S_{k|k-1}^{-1}(y_{dk}-d_{k})\\ P_{k|k}=P_{k|k-1}-\psi_{k|k-1}S_{k|k-1}^{-1}\psi_{k|k-1}^{T} \end{array}\right.$$

where *d_k_* is the measurement output of the system at the *k* moment, *y_dk_* is the expected trajectory of the system at the *k* moment, and the (*y_dk_ − d_k_*) is used as the new information of UKF.

The feedback control voltage *u_fb_* is:

(21) $$u_{fb}=H(x_{k|k})$$

Ultimately, the control input to the VA-PEA module is:

(22) $$\begin{array}{ll} u & =u_{ff}+u_{fb}\\ & =sat\left(H_{\mathrm{PI}}(y_{dp})\right)+K_{P}e+x\int edt \end{array}$$

## 4. Experimental Verifications

To verify the effectiveness of the proposed feedforward–feedback combined control, trajectory tracking experiments are conducted on the PEA. For the purpose of comparison, the following control methods are considered, i.e., pure feedforward with phase–hysteresis hybrid compensation (labeled as FF), pure feedback using a PID controller (labeled as FB), feedforward–feedback combined control with phase–hysteresis hybrid compensation and a PID controller (labeled as FF + PID), and the proposed feedforward–feedback combined control with phase–hysteresis hybrid compensation and a UKF-based PID controller (labeled as FF + PID + UKF). In FB and FF + PID, for the gains of the PID controller, the proportional gain is roughly the ratio of voltage to displacement, i.e., 0.8 in this paper. The integral gain is set to 500 after a trial and error process. As previously stated, the derivative gain is set to 0 as the closed-loop controller is only responsible for the modeling uncertainties. In FF + PID + UKF, the initial proportional and integral gains are also set to 0.8 and 500, respectively.

**Figure 9:**
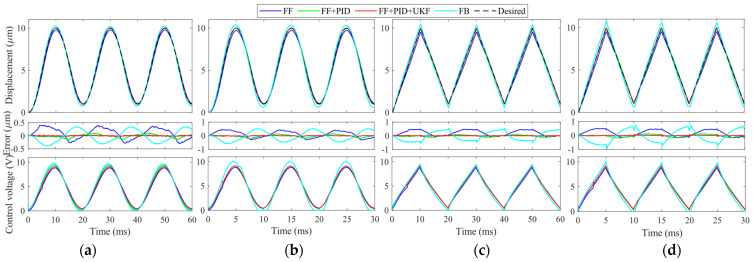
Hysteresis compensation results and control voltage *u* of signals at low frequencies. (**a**) 50 Hz sinusoidal trajectory; (**b**) 100 Hz sinusoidal trajectory; (**c**) 50 Hz triangular trajectory; and (**d**) 100 Hz triangular trajectory.

**Figure 10:**
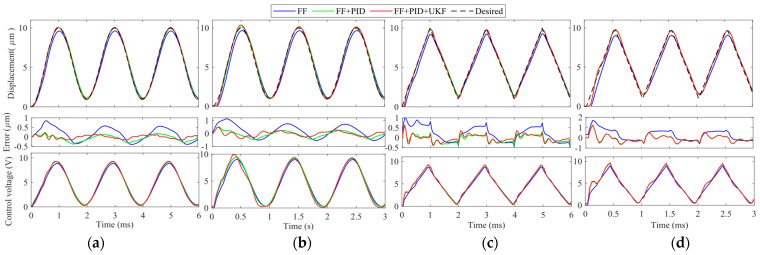
Hysteresis compensation performances and control voltage *u* of signals at high frequencies. (**a**) 500 Hz sinusoidal trajectory; (**b**) 1000 Hz sinusoidal trajectory; (**c**) 500 Hz triangular trajectory; and (**d**) 1000 Hz triangular trajectory.

**Figure 11:**
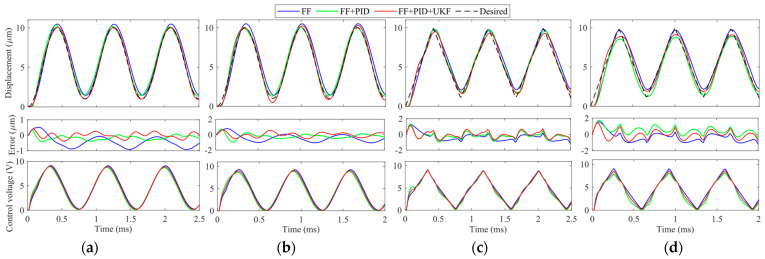
Hysteresis compensation performances and control voltage *u* of signals above 1000 Hz. (**a**) 1200 Hz sinusoidal trajectory; (**b**) 1500 Hz sinusoidal trajectory; (**c**) 1200 Hz triangular trajectory; and (**d**) 1500 Hz triangular trajectory.

The maximum input voltage is kept below 100 V to avoid possible excess of the PEA’s voltage limit. The experiments can be divided into the following three categories:

(1) **Low-frequency tracking:** Sinusoidal and triangular trajectories at 50 Hz and 100 Hz are adopted as the desired trajectories. The trajectory tracking results are provided in Figure 9. For these two low-frequency trajectories, the influences of the linear dynamics of the VA-PEA module are not significant. All control methods can successfully compensate for the hysteresis of the VA-PEA module. Except for FB, the PEA can follow the desired trajectory with small distortions. It can be seen that even in FF, the hysteresis compensation performance is satisfactory. This verifies the modeling accuracy of the inverse PI model. If a feedback controller is also integrated with FF, the hysteresis compensation performance can be further improved. For the proposed FF + PID + UKF, it can be seen from the error plots that the tracking error is very close to 0, showing an excellent hysteresis compensation performance. It can be found that the control voltage *u* stays well between 0 and 10 V.

(2) **High-frequency tracking:** The frequency of the desired trajectory is increased to 500 Hz and 1000 Hz so as to test the high-frequency performance of the proposed FF + PID + UKF. The experimental results are provided in Figure 10. Similar to the results in Figure 9, in high-frequency experiments, the feedforward compensation is also very good. After adding a feedback controller, the tracking error can be further reduced. The proposed FF + PID + UKF achieves the best performance among the controllers. However, it is found that it is very difficult to tune the gains of the PID in FB. As a result, FB is not included in Figure 10.

(3) **Trajectory tracking for frequencies above 1000 Hz:** In order to further test the high-frequency trajectory tracking capability of the proposed method, the frequency of the desired trajectory is increased to 1200 Hz and 1500 Hz. The experiment results are provided in Figure 11. Due to the high modeling accuracy of the phase–hysteresis hybrid compensation, the dynamics and hysteresis of the VE-PEA can also be suppressed in these two high-frequency trajectories. Similar results to Figure 9 and Figure 10 can also observed.

In order to quantitively compare the hysteresis compensation and trajectory tracking performances of these control methods, the root mean square error (RMSE) and the mean absolute error (MAE) are calculated and listed in Table 3. It can be seen that the proposed method exhibits the best performance in all experimental groups. The RMSE and MAE of the proposed method in each group of experiments are the smallest.

**Figure 12:**
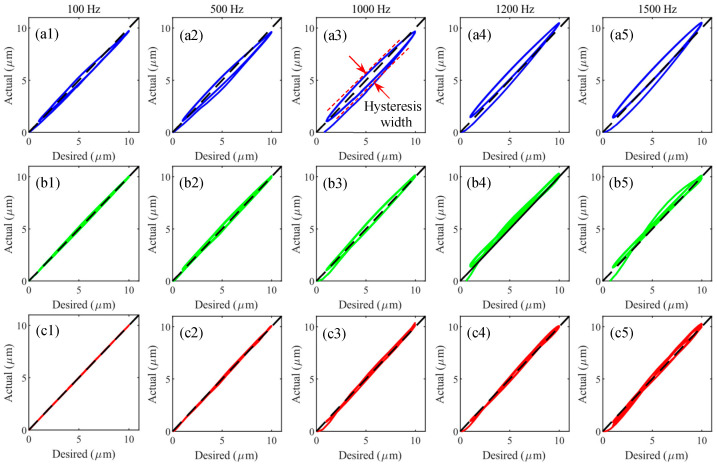
The desired–actual curves of the compensated system in tracking sinusoidal trajectories. (**a1**–**a5**) The results of FF, (**b1**–**b5**) the results of FF + PID, and (**c1**–**c5**) the results of FF + PID + UKF.

For the hysteresis compensation, the relationships between the desired and actual trajectories of the compensated system in tracking sinusoidal trajectories are obtained and presented in Figure 12. A 45° line is also plotted in each figure to indicate the unitary mapping from the desired trajectory to the actual trajectory. The closer to the 45° line, the better hysteresis compensation performance. At frequencies below 500 Hz, the desired–actual curves of the proposed FF + PID + UKF almost coincide with the 45° line, showing excellent hysteresis compensation performance. For frequencies higher than 500 Hz, the desired–actual curves of the proposed FF + PID + UKF also stay the closest to the 45° line when compared with the other methods. The experimental results in tracking triangular trajectories are similar and thus will not be presented.

In order to quantitatively assess the hysteresis compensation performance, the hysteresis width of the closed-loop system is proposed. As shown in Figure 12a3 for the closed-loop system, the 45° line indicates the unitary mapping from between the desired and actual trajectories. As a result, the width of the input–output curve perpendicular to the 45° line is defined as the hysteresis width. This index is reasonable for hysteresis compensation as the hysteresis width will converge to zero if the hysteresis is successfully compensated for. The hysteresis widths of the closed-loop system using different controllers are listed in Table 4.

**Figure 13:**
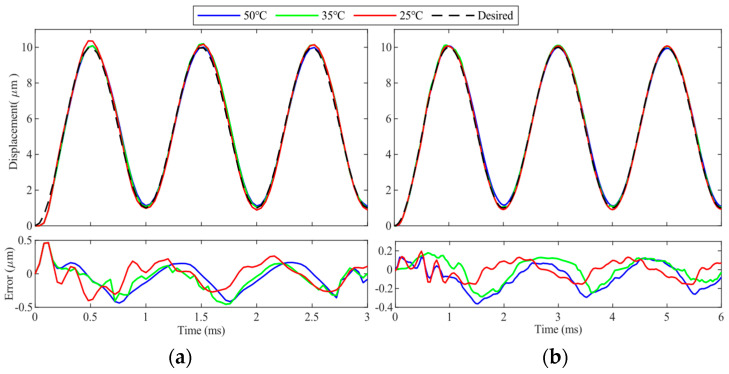
Trajectory tracking performances of the proposed method at environment temperatures of 25 °C, 35 °C, and 50 °C. (**a**,**b**) 500 Hz and 1000 Hz sinusoidal trajectories.

It must be pointed out that the PEA is also temperature-sensitive. To verify the effectiveness of the proposed method at different environment temperatures, tracking of 500 Hz and 1000 Hz sinusoidal trajectories is conducted at environment temperatures of 25 °C, 35 °C, and 50 °C, respectively. Because the maximum allowable environment temperature of the PEA selected in this paper is 85 °C, the environment temperature is maintained below 50 °C for the safety of the experiment. The experimental results are provided in Figure 13, and the root mean square errors are calculated and shown in Table 5. It can be observed that the trajectory tracking performance of the proposed method only varies slightly at different temperatures. Therefore, it can be concluded that the proposed method is also effective in different temperatures.

## 5. Conclusions

In this paper, a feedforward–feedback combined control based on phase–hysteresis hybrid compensation is proposed to compensate for the coupled dynamics and hysteresis of the VA-PEA module. In phase–hysteresis hybrid compensation, a lead compensator is constructed to account for the phase lag of the linear dynamics of the VA-PEA module, and an inverse PI model is used to compensate for the hysteresis of the PEA. In this manner, the influence of the dynamics and hysteresis in high-frequency manipulations can be significantly compensated for. In the feedback loop, a UKF-based PID controller with a self-tuning capability is used to further compensate for the modeling uncertainties of the phase–hysteresis compensation.

Tracking of sinusoidal and triangular trajectories at frequencies up to 1500 Hz is implemented. The experimental results have shown that using phase–hysteresis hybrid compensation, the majority of the dynamics and hysteresis of the VA-PEA module can be well compensated for. As a result, the trajectory tracking and hysteresis compensation performance can be efficiently improved under this feedforward–feedback combined control scheme. Compared with the conventional trajectory tracking at the level of 200–300 Hz, the closed-loop trajectory tracking bandwidth can be increased to 1500 Hz using the proposed method.

## Figures and Tables

**Table 1:** Parameters of the modified PI model.

	1	2	3	4	5	6	7	8	9	10
*r_i_*	0	3.7101	21.1438	32.1438	40.5758	45.1003	47.1873	69.81	81.5687	90.6485
*w_hi_*	8.437 × 10^−3^	2.851 × 10^−3^	7.517 × 10^−4^	1.856 × 10^−9^	3.019 × 10^−3^	−0.0179	0.0140	1.695 × 10^−3^	2.404 × 10^−3^	−0.2041
*c_i_*	8.8816	/	2.5262	/	−2.9198	/	0.6430	/	0.1342	/

**Table 2:** Parameters of the inverse PI model.

	1	2	3	4	5	6	7	8	9	10
$r_{i}^{-1}$	0	0.1306	3.0631	3.9282	5.5533	4.9691	5.5667	7.6329	9.4221	10.9181
$w_{hi}^{-1}$	1.4874	−0.3781	−0.1677	−0.1395	4.9229	−0.0918	−4.6767	−0.1370	−0.0595	−0.0485
$c_{i}^{-1}$	3.700 × 10^−9^	/	−35.166	/	7.045 × 10^−4^	/	−0.0462	/	9.4131	/

**Table 3:** Statistics on the hysteresis compensation and trajectory tracking performances.

Trajectory	Controller	RMSE (μm)/MAE (μm)					
		50 Hz	100 Hz	500 Hz	1000 Hz	1200 Hz	1500 Hz
Sinusoidal	FB	0.2311/0.2042	0.3466/0.3062	/	/	/	/
	FF	0.2255/0.1972	0.2605/0.2308	0.3783/0.3268	0.5178/0.4405	0.5630/0.4912	0.6391/0.5339
	FF + PID	0.0582/0.0663	0.0880/0.0694	0.1691/0.1414	0.2452/0.2064	0.2124/0.1785	0.3446/0.2699
	FF + PID + UKF	0.0096/0.0078	0.0172/0.0141	0.0909/0.0764	0.1893/0.1583	0.1813/0.1473	0.2914/0.2420
Triangular	FB	0.3312/0.3059	0.4497/0.4030	/	/	/	/
	FF	0.2846/0.2260	0.2890/0.2423	0.4313/0.3665	0.5957/0.4910	0.5981/0.5025	0.6980/0.5713
	FF + PID	0.0587/0.0485	0.0884/0.0755	0.2035/0.1761	0.3932/0.3136	0.4393/0.3464	0.6596/0.5356
	FF + PID + UKF	0.0113/0.0087	0.0196/0.0146	0.1842/0.1548	03539/0.2816	0.3821/0.3189	0.5118/0.4011

**Table 4:** The hysteresis width using different control methods. (Unit: μm).

	100 Hz	500 Hz	1000 Hz	1200 Hz	1500 Hz
FF	0.5400	0.8852	1.1889	1.2198	1.2992
FF + PID	0.2488	0.4396	0.6982	0.7096	1.2121
FF + PID + UKF	**0.0644**	**0.2492**	**0.6124**	**0.6264**	**0.8146**

The bold is used to make the results of the proposed method clearer.

**Table 5:** Root mean square errors at different temperatures. (Unit: μm).

	25 °C	35 °C	50 °C
500 Hz	0.0909	0.1172	0.1361
1000 Hz	0.1893	0.1968	0.2034

## Data Availability

The data that support the findings of this study are available from the corresponding author upon reasonable request.
